# Breast implant iatrogenics: challenging the safety narrative

**DOI:** 10.3389/fgwh.2024.1359106

**Published:** 2024-05-17

**Authors:** Siham Azahaf, Karlinde A. Spit, Christel J. M. de Blok, Laura Willging, Heidi Rolfs, Prabath W. B. Nanayakkara

**Affiliations:** ^1^ Section General Internal Medicine, Department of Internal Medicine, Amsterdam University Medical Centers, Location VUmc, Amsterdam, Netherlands; ^2^ Board of Directors, Breast Implant Safety Alliance (BISA) non-Profit, Charleston, SC, United States

**Keywords:** breast implants, breast implant safety, iatrogenics, women’s health, public health, breast augmentation, implant complications, medical device safety

## Introduction

Since their 1960s debut, silicone breast implants are a subject of ongoing safety debates. Their “grandfathered” status under the 1976 Medical Device Amendments resulted in a lack of pre-market safety data. In 1992, the FDA imposed a 14-year long ban due to escalating safety concerns over reported health complaints by women. The ban was lifted after a subsequent review by the Institute of Medicine concluded that breast implants do not cause major illnesses, such as cancer (1). Despite their approved re-entry to the market and widespread use, safety concerns regarding these high-risk classified devices persist and have intensified. This viewpoint challenges the prevailing safety narrative, highlighting persistent device failure and implant-associated phenomena, urging comprehensive re-evaluation of their use.

### Implant failure

The evolution of silicone breast implants has resulted in over 8,300 distinct implant variations (1). Nonetheless, device failure remains of significant concern today (2). Within a 15-year time frame, approximately half of the women with silicone breast implants may experience implant failure, with 10% occurring within the first two to four years post-implantation (3, 4).

Prevalent implant complications include ruptures and capsular contraction. Rupture rates vary from 1% to 35% within a decade, yet remain challenging to accurately determine due to inconsistent screening practices (3, 5, 6). Timely detection of silicone leakage, due to ruptures or extensive bleeding from intact implants, is crucial to mitigate the risks of unpredictable silicone migration, as exemplified by disturbing case reports. Migrating silicone particles can induce inflammation leading to silicone-induced lymphadenopathy (7), acute respiratory distress syndrome (8, 9), chronic pulmonary embolism (9, 10), sarcoidosis (11) and scleroderma of the skin (12). Granuloma formation may occur (13), potentially resulting in embolism formation and hematological spread to, for example, the carotid artery leading to ocular muscle palsy (14). Migration of silicone particles to distant organs can also occur from intact implants (15–17). The spread and potential damage is often irreversible due to the inability to eliminate silicone particles from the body. Consequently, individuals who remain asymptomatic despite silicone migration may experience complications later in time. While the FDA prohibits the use of injectable silicone for body contouring due to the significant risks of silicone migration, the use of breast implants is permitted because the silicone is contained within a shell. However, as presented above, similar risks are observed when silicone particles bleed from intact implants or spread from ruptured implants (18).

Attempts to modify implant characteristics, including the use of double layers to reduce rupture rates or the incorporation of corticosteroids in the implants and the (macro)texturing of the surface to minimize capsular contracture, have been proven insufficient in effectively mitigating these risks (1). It has been estimated that a 41-year old woman undergoing initial implantation may anticipate a total of four additional implant surgeries by the age of 85 (19).

In fact, new risks were introduced since (macro)texturing of the implant surface is a recognized risk factor for Breast Implant Associated-Anaplastic-Large Cell Lymphoma (BIA-ALCL) (20). One could argue that the recurring intervention cycle of designing a newer generation breast implant and introducing new risks paradoxically impedes innovation, constraining the exploration of safer and more sustainable alternatives to silicone breast implants.

The FDA currently advises women to undergo regular screenings for silent ruptures every 2–3 years and preventive routine replacements every 10–15 years (21). However, adherence is poor due to apprehension about potential health consequences and the financial burden related to both screening and surgeries (22). Women are thereby subjected to physical, mental and financial risks associated with the implants and repeated surgical procedures.

### Implant epiphenomena

The recent discovery of Breast Implant-Associated Squamous Cell Carcinoma (BIA-SCC), sarcoma and other lymphomas extends safety concerns beyond device failure. The six-month mortality rate for BIA-SCC, estimated at 44%, is particularly distressing (23). Despite their presumed rarity, underreporting due to under recognition may lead to underestimation of the true global incidence, as exemplified by the delay in identifying breast implant-associated anaplastic large cell lymphoma (BIA-ALCL). The rising number of cases and deaths attributed to BIA-ALCL illustrates this issue, with 1,358 cases as of April 1, 2024 and 59 deaths up to April 2022 (24). In a global survey of 628 plastic surgeons, 2%–14% reported encountering BIA-ALCL at least once (25). This implies that if roughly 8% of the plastic surgeons across the top 20 countries with the highest number of plastic surgeons worldwide encountered BIA-ALCL at least once, we would anticipate approximately 3,424 cases at present, exceeding our current estimates by more than 50% (26).

It can be argued that the best risk reduction technique would be to avoid exposure to a causative factor. However, apart from a few nations like France with a total ban on all textured implants, the use of (macro)textured implants remains unrestricted (27). Controversy persists as some plastic surgeons prefer textured implants for their reported benefits, such as reduced risk of capsular contracture (28–30). Prophylactic removal of macrotextured surface implants has recently been considered reasonable by the American Association of Plastic Surgeons (31, 32). However, it remains unknown if implant removal reduces the risks of any implant related cancer (33). The FDA responded that prophylactic removal is not recommended in asymptomatic individuals due to BIA-ALCL being uncommon (34).

The paucity of reported cases of breast implant-related cancers should not engender complacency, as the small number of observations introduce substantial uncertainty in estimating their probability. In fact, this is illustrated in the current incidence estimates of BIA-ALCL, which range from 1 in 355 to 1 in 30,000 patients, depending on patient and implant characteristics (35–39). Current risk assessment methods have proven insufficient in capturing low-probability yet high-impact events, as demonstrated by the decades-long delay in identifying the initially dismissed implant-related cancers. This underscores the urgency of transitioning from merely assessing occurrence probability to considering impact and implementing preemptive risk mitigation strategies.

### Breast implant illness

Reports of unexplained systemic symptoms attributed to silicone breast implants have been consistent since their introduction. This constellation of symptoms is referred to as Breast Implant Illness (BII), among other nomenclatures (40). The symptoms include, but are not limited to, debilitating fatigue, joint pains, and night sweats. The high incidence (75%) of reported concomitant local complaints suggest an interplay in the onset of BII (41). While the pathophysiology remains to be elucidated, the main proposed mechanism of disease is silicone- induced inflammation (42). The heterogeneity in the presentation of BII suggests complex interactions of internal and external factors that have yet to be identified. The assertion of causality has been supported using Bradford Hill Criteria (40). While the FDA included the notion of breast implant-related systemic symptoms in their black box warning, BII is still being denied by many physicians, leading to bias and consequent underrecognition and underreporting of BII (43, 44).

Clinical management of BII is hampered by the absence of definitive diagnostic criteria and diagnostic biomarkers, complicating decision-making regarding implant removal. Additionally, the lack of reliable incidence numbers complicate risk assessment. Drawing upon our experience at the silicone outpatient clinic at Amsterdam University Medical Centers, where we have observed, treated, and studied over 2,000 women with BII, we approach BII also as a *diagnosis per exclusionem.* We found that 2 out of 3 women reported improvement of symptoms after surgical removal of the implants (41). In addition, our experience suggests that women with substantial silicone residues are less likely to show improvement of symptoms, a trend supported by a study where women with extracapsular silicone were more 2.8 times more likely to report a diagnosis with fibromyalgia (45). It remains unclear what potential risks are of silicone residues in the human body. While our current understanding suggests that only a subset of women may be susceptible to BII, there is currently no reliable method to predict who is at risk. Nevertheless, BII can have a profound impact on the quality of life, leaving a subset of women unable to participate in daily activities.

The link between silicone breast implants and established autoimmune disease (AID) remains contentious, marked by conflicting evidence (42). This may be attributed to the atypical presentation of breast implant-related AID which may not invariably align with diagnostic criteria for established AID. This is illustrated by a case report from our clinic on silicone induced scleroderma with an atypical presentation, and underscores the complexity of diagnosing AID in the context of breast implants (12). Furthermore, the inherently low incidence of AID in the general population suggests an even lower likelihood of diagnosing breast implant-induced AID, which often goes unrecognized. Cohort studies are generally not well-suited for detecting rare diseases. However, several cohort studies found significant higher risk of AID in women with breast implants, with the most notable being Sjögren's syndrome, systemic sclerosis, and sarcoidosis (46, 47).

### Implant conundrum

Breast implant related risks cause significant apprehension among implant recipients (48). In the US, removal of implants for aesthetic purposes is generally not covered by insurance. Consequently, women experiencing health complaints attributed to the implants, or women who were inadequately informed about associated risks during implantation that wish for surgical removal of the implants, are burdened with financial barriers.

The limited availability of insurance coverage is a notable concern in the context of breast implant-related cancers. While breast reconstruction patients receive insurance coverage for the treatment of BIA-ALCL, a study found that only 22% of women with implants for cosmetic purposes were initially covered. Seven percent of women diagnosed with BIA-ALCL were denied insurance coverage even after two appeals from their physicians (49).

Moreover, women that choose to undergo an “aesthetic flat closure” following a mastectomy encounter unsupportive healthcare environments (50). This issue, referred to as “flat denial”, arises when a surgeon either discourages or neglects to provide a flat closure, or leaves excess skin for potential future reconstruction against the patient's wishes. One study reported 22% of women to experience flat denial (51). Aesthetic flat closures offer a viable alternative to silicone breast implants and should be discussed as an option with patients. Notably, two studies have reported high satisfaction rates of 84% and 71% for aesthetic flat closures (51, 52).

### Risk-Benefit asymmetry

While all medical devices carry some risk of failure, many provide substantial health benefits, creating a favorable risk-benefit asymmetry. For example, hip replacements are commonly implanted in older patients to substantially restore function, typically requiring replacement only once. However, breast implant's benefits are mainly aesthetic and psychological and thereby comparatively less substantial. The majority of breast implants are implanted in healthy young individuals, extending the exposure time to risks. Patients receiving breast implants after reconstruction have an even higher risk of device failure and associated risks (53). Furthermore, the prevalence of BIA-ALCL is conjectured to be higher in patients with genetic predisposition to breast cancer (54). The risks are not linear but rather exhibit a convex response, accelerating with prolonged exposure. This underscores the importance of device safety, particularly when dealing with the human body—a complex and dynamic system where significant adverse outcomes are often unpredictable.

### Turning tables

In the annals of medical history, the pursuit of innovation has often led to unforeseen repercussions. The predominant narrative of assumed breast implant safety is challenged by substantial device failure, emerging implant-related cancers, and poorly understood BII symptoms, skewing the risk-benefit equation. It becomes prudent to apply the precautionary principle when assessing the trade-off between the benefits and the risks associated with breast implants. This principle asserts that in the absence of scientific consensus, the burden of proof falls on those advocating for a policy or action that could cause harm to the public. Our argument advocates for public safety prioritization, pressing all regulatory bodies to re-evaluate breast implant safety and promote the exploration of safer alternatives.
